# Puerperal Eclampsia

**Published:** 1878-03

**Authors:** J. W. Griggs

**Affiliations:** West Point, Ga.


ATLANTA
MEDICAL AND SURGICAL JOURNAL

VOL. XV.] MARCH—1878. [NO. 12

### Original Communications.

### THE FOURTH CASE OF PUERPERAL ECLAMPSIA SUCCESSFULLY TREATED BY THE HYPODERMIC USE OF VERATRUM VIRIDE, IN ANSWER TO THE PRIORITY CLAIM OF W. A. GREENE, M.D., MACON, GA.

BY J. W. GRIGGS, M.D., WEST POINT, GA.

The January number of the Atlanta MEDICAL AND SURGICAL JOURNAL, for 1877, contained an article submitted by me, reciting three cases of puerperal eclampsia which I had successfully treated by the hypodermic use of the tincture of veratrum viride. It is now my pleasure to report the fourth.

On Monday, February 25, 1878, I was summoned to see a negress, a primipara, æt. fifteen years, living on the plantation of Mr. Benj. Anderson, in Chambers county, Ala., five miles distant. I arrived at 2½ o'clock A.M., and found her having convulsions every fifteen minutes. She was very stout and considerably swollen, her face and extremities being quite œdematous. There were no symptoms of labor, unless we esteem the eclampsia in such cases to be distributed or misdirected labor. I was informed that she had complained very much of a pain over the crown of the head for several days, and that convulsions had come up the evening before, about sundown. The interval had gradually diminished until fifteen minutes only elapsed between paroxysms. She had been unconscious since early in the night, and was now altogether insensible, frothing at the mouth, with loud, stertorous respiration, the expiratory act accompanied by the greatest noise. The pulse was very full and hard, at about ninety beats per minute. By the time that I had acquainted myself with the facts, a terrible convulsion came up. I now determined to test the unaided powers of the veratrum viride, and, as soon as the spasm passed off, introduced subcutaneously gtt. ij of the tincture. In less than five minutes the convulsion was repeated, and, when passed, gtt. ij were administered. Fifty minutes elapsed, and eclampsia returned more fearfully than ever. As soon as this was over I injected gtt. iv and she slept quietly for two hours, then another convulsion occurred. I introduced again gtt. iv, and the pulse soon went down to fifty-six beats per minute, and I stood sentinel over it, injecting subcutaneously sometimes three drops and sometimes two drops every two or three hours, as the symptoms seemed to require. The treatment was kept up until twelve hours had been passed without a return. I never allowed the pulse to rise above sixty. The patient had received no other treatment up to this time, except the introduction of $\frac{1}{8}$ gr. morph. sulp. with the veratrum, at eleven o'clock A.M., which was given to allay the intense thirst of the patient and to subdue the nausea, which was only slight. This was suggested by my father, Dr. A. W. Griggs, of this place, who came out at this juncture to see how I was getting on. She became more and more conscious every hour after the eclampsia ceased, and now recognized us all, but seemed astonished at our presence. She had frequently called for water for the last hour or two, and now was induced to pass urine for the first time in fifteen hours. She voided over half a pint, and it yielded the greatest per centum of albumen I ever knew.

No symptom of labor having appeared, we succeeded the last hypodermic dose of veratrum, in three hours, by three drops of the same per orem, and leaving her in charge of an intelligent nurse, supervised by Mr. Anderson, we directed that three drops of veratrum be given every three hours, and fifteen drops of chloroform an hour and a half after each dose of veratrum. Thus we determined to keep down the eclampsia and counteract or remove the immediate cause. The instructions to the nurse being well explained we returned home, expecting to be called if anything should go wrong, or if labor should supervene. The next morning we learned that she had passed a tolerably comfortable night, and would have rested better but for the pain over the crown of the head, which was now some better. On Tuesday night they sent in for me, and, arriving, I found that she had had transient pains before noon, which soon stopped, but that labor set in about night.

The treatment had been continued. The pulse being rather weak, the veratrum was now omitted, and the chloroform continued; labor was well nigh completed, and a well developed living male child was born Wednesday morning at three o'clock. The chloroform was now discontinued, and the patient did well until the next evening, when some disturbance occurred in the sensory system—headache and blindness—and, although the urine yielded now but a comparatively small per centum of albumen, the chloroform was again given, and continued twenty-four hours, when all the unpleasant symptoms subsided, and it was discontinued. The patient has been doing well ever since.

Now, I think that this is the first case of puerperal eclampsia ever treated by the veratrum viride, unaided and alone. If I am wrong please inform me.

After I had published my other cases, Dr. W. A. Greene, of Macon, Ga., sent a very courteous communication to the February number of your JOURNAL for 1877, calling me to an account as to priority in the mode of using the tincture of veratrum in puerperal eclampsia. If the doctor had read me closely, he would not have made the mistake. If he will refer to the article, he will see that in my first case, on March 5th, 1875, I at first administered hypodermically $\frac{1}{6}$ gr. of morphine without results. Then, after an interval, the convulsions continuing, I inserted gtt. ij. of veratrum viride with morphia sulph., gr. $\frac{1}{8}$, with needle syringe, which produced a marked effect, although an abortive spasm shortly afterwards came on, and she then rested quietly for two hours, when three drops of veratrum tincture were injected, producing the desired sedative effect. "The treatment consisted in keeping the circulation a little below the normal pulse." The doctor will now see that I relied entirely on veratrum after I began its use, and that the remedy was so administered as to keep the circulation below the normal standard.

In case second, the patient had been bled before I saw her—had taken chloral hydrate, bromide of potassium and cold affusions to the head. "There was no abatement of symptoms from the treatment." "The pulse was 100 to 120. We then inserted by hypodermy, morphia sulph. gr. $\frac{1}{6}$, tincture veratrum viride gtt. iij. The pulse sank in thirty-five minutes to 90, and she did not have a convulsion in two hours and a half. Then we noticed an increase in the pulse before the attack came on, which was not so severe as former paroxysms had been. After this, the circulation was closely watched, and the pulse was kept below 70 beats per minute, by the occasional introduction of two or three drops of veratrum, hypodermically." Now, did I not rely entirely on the veratrum in this case?

Case third will show the same confidence on my part. "Before the veratrum was used, the attacks were every thirty or forty minutes; after its use the interval was prolonged to five hours." "There was but little albumenuria in this case." In the concluding paragraph I said: "My father has long been in the habit of using veratrum in puerperal and hysterical convulsions, but I think I am the first to use it in the way described in this paper." Perhaps my language was unfortunate, for although I had never seen Dr. Greene's paper on "Hypodermic Medication," I was aware that my father and others had frequently used veratrum hypodermically. The veratrum, in such cases, was thought a valuable addition to other treatment, and its effects mostly auxiliary. But I used it as the *sine qua non ad rem* for eclampsia.

I would have replied to Dr. Greene before, for I have a high regard for his opinions, but determined not to go before the profession without presenting something for consideration tending to the progress of medical science; so I patiently waited for another case before speaking about the priority claim. I would not detract from the honor or glory of any man battling for science or humanity. I would rather give the helping hand. Dr. Greene's high reputation as a physician, and his devotion to the profession, command the greatest respect, and I hope that he will understand me. His paper on "Hypodermic Medication" is certainly a valuable addendum to therapeutics. He speaks highly of the effects of veratrum administered hypodermically in two cases of puerperal eclampsia. Indeed, he speaks of it as a "most valuable remedy." In his first case he bled until the "pulse had sunk very low." The interval, which had been from twenty to forty minutes between attacks, was now lengthened to about an hour. Then a violent eclampsia came on, and when passed he administered "ten drops of the solution of morphine and atropine, which had the effect to quiet her at once." Another convulsion came on in about an hour and forty minutes. He then injected gtt. ij. of veratrum, which was repeated in fifteen minutes, gtt. iij. being used; afterwards gtt. iv. were used, and the eclampsia ceased. The patient was put on ʒ ss. doses of the potassium bromide every six or eight hours. The circulation was kept under control for two or three days by occasional doses of veratrum, combined with bromide.

In his second case, the convulsions had been coming on every ten or fifteen minutes, until Dr. Hudson arrived about an hour after she had been taken, and he gave her "a large dose of morphine, and put her under chloroform, when the convulsions were delayed an hour or more in their intervals," but again became rapid. On Dr. Greene's arrival, the chloroform was withdrawn, and he "bled her eighteen ounces, and gave her ten drops of solution of morphine and atropia fifteen minutes thereafter; had five convulsions in quick succession, the last one lightest. At 11½ o'clock, A.M., pulse 145; injected one drop of veratrum in a few drops of water. In ten minutes pulse reduced to 130; in twenty minutes to 120; 12¾ a convulsion; eight minutes to 1 P.M., gave one drop veratrum combined with eight drops solution morphine and atropia; pulse 115; 1:25 P.M., pulse 135, and convulsions came on; bled her twelve ounces; 1:32 injected gr. ss. ergotine, combined with gtt. ij. of veratrum in thigh, pulse being 135. In eighteen minutes pulse reduced to 120; twenty minutes no change; twenty minutes more pulse 110; 1½ convulsion; 3:7 pulse 128; os uteri yet contracted and no uterine pain, coma being profound and stertorous, rapid breathing; child alive. Three P.M. ruptured membranes, and injected half grain of ergotine in thigh, combined with three drops of veratrum; 3:45, pulse 120; injected three drops of veratrum, in ten minutes pulse 116, and gave half grain ergotine; some uterine contractions; waters all passed off; os dilated size half dollar; 5 P.M. severe convulsions; 5:40 half grain ergotine injected in abdomen; uterine contractions decidedly increased; coma very profound; 6:45 P.M. convulsions; uterine contraction going on; 7 P.M head of fœtus descended so that forceps could be applied, which was done and a twelve pound child extracted.

Now, it appears that this patient was bled thirty ounces—eighteen at one time and twelve at another—and that morph. and atropia were also used, and afterwards veratrum; again, veratrum, morphine and atropia, the last named being a powerful remedy.

This and the former case were not treated as I treated any of my cases; for my dependence for relief was in the veratrum, and my fourth case, with eighteen convulsions, proves that the veratrum is equal to the emergency in cases of puerperal eclampsia, caused by albumenuria. I will discuss the *modus operandi* of the veratrum tincture in these cases in a future paper.

